# The prognostic value of GLUT1 in cancers: a systematic review and meta-analysis

**DOI:** 10.18632/oncotarget.17445

**Published:** 2017-04-27

**Authors:** Min Yu, Han Yongzhi, Shengying Chen, Xiaodan Luo, Ye Lin, Yu Zhou, Haosheng Jin, Baohua Hou, Yanying Deng, Lei Tu, Zhixiang Jian

**Affiliations:** ^1^ Department of General Surgery, Guangdong General Hospital, Guangdong Academy of Medical Sciences, Guangzhou, China; ^2^ Department of Dermatology, Guangdong General Hospital, Guangdong Academy of Medical Science, Guangzhou, China; ^3^ Department of Infectious Diseases, Guangdong General Hospital, Guangdong Academy of Medical Science, Guangzhou, China

**Keywords:** glucose transporter 1, prognostic marker, survival, cancer, glycolysis

## Abstract

Increased glycolysis is one of the hallmarks of cancer. The abnormal expression of glucose transporter 1 (GLUT1) was reported to be associated with resistance to current therapy and poor prognosis. Numerous studies have investigated the correlation between GLUT1 expression and prognosis in cancers, but the conclusions are still controversial. Here, we conducted a meta-analysis to explore the association between GLUT1 and survival in human cancers. PubMed, Springer, Medline, and Cochrane Library were searched carefully to identify eligible studies evaluating prognostic value of GLUT1 in cancers. Twenty-seven studies with 4079 patients were included in the present study. Our pooled results identified that increased expression of GLUT1 was associated with unfavorable overall survival (HR = 1.780, 95% CI = 1.574–.013, *p* < 0.001)) and poorer disease-free survival (HR = 1.95, 95% CI = 1.229–3.095, *p* = 0.003). Furthermore, overexpression of GLUT1 linked with poor differentiated tumors (RR = 1.380, 95% CI = 1.086–1.755, *p* = 0.009; I^2^ = 72.0%, *p* < 0.001), positive lymph node metastasis (RR = 1.395, 95% CI = 1.082–1.799, *p* = 0.010; I^2^ = 70.8%, *p* = 0.002) and larger tumor size (RR = 1.405, 95% CI = 1.231–1.603, *p <* 0.001; I^2^ = 37.3%, *p* = 0.093). This systematic review and meta-analysis indicated that the GLUT1 may serve as an ideal prognostic biomarker in various cancers.

## INTRODUCTION

Tumor cells exhibit an altered metabolism to support their fury proliferation under robust environment. Increased need for glycolysis, known as Warburg effect, and glucose uptake for energy production were identified in various cancers [1]. Although oxidative catabolism was more efficient in ATP production, glycolysis was identified increased along with upregulation of glucose transporters. Recent studies have showed that overexpression of glucose transporters (GLUTs), a protein family responsible for glucose uptake, resulted in enhanced aerobic glycolysis of cancers [2]. To date, 14 members of the glucose transporter family have been reported. Based on the sequence of similarities and structure elements, the glucose transporter family can be divided into three subfamilies [3]. Among them, GLUT1, encoded by the SLC2A1 gene, is likely one of the most extensively studied proteins of all membrane transport systems. GLUT1 is a representative protein of GLUT family and is widely distributed in normal tissues. GLUT1 is primarily undetectable in normal epithelial tissues and benign epithelial tumors. Overexpression of GLUT1 during the oncogenesis has been identified in various cancers, which results in increased glucose uptake into cytoplasm of tumor cells [4].

Given the importance of GLUT1 in oncogenesis, some studies were conducted to investigate the prognostic value of GLUT1 in tumors. However, conflicting results were found across different tumors. Some studies reported that overexpression of GLUT1 was significantly associated with poor survival in patients with different cancers, whereas others found no significant association [4]. Identification of patients with poor prognosis can help develop novel treatment strategies at the beginning of treatment, which may lead to better and more individual therapy strategies with superior survival. Therefore, it is meaningful to further evaluate the prognostic value of GLUT1 in cancers. Here, we conducted a systematic review and meta-analysis of published literature to investigate and determine the prognostic value of GLUT1 among different cancers and to provide objective evidence to support further prospective clinical studies.

## RESULTS

### Study selection and description of the include studies

**Figure 1:**
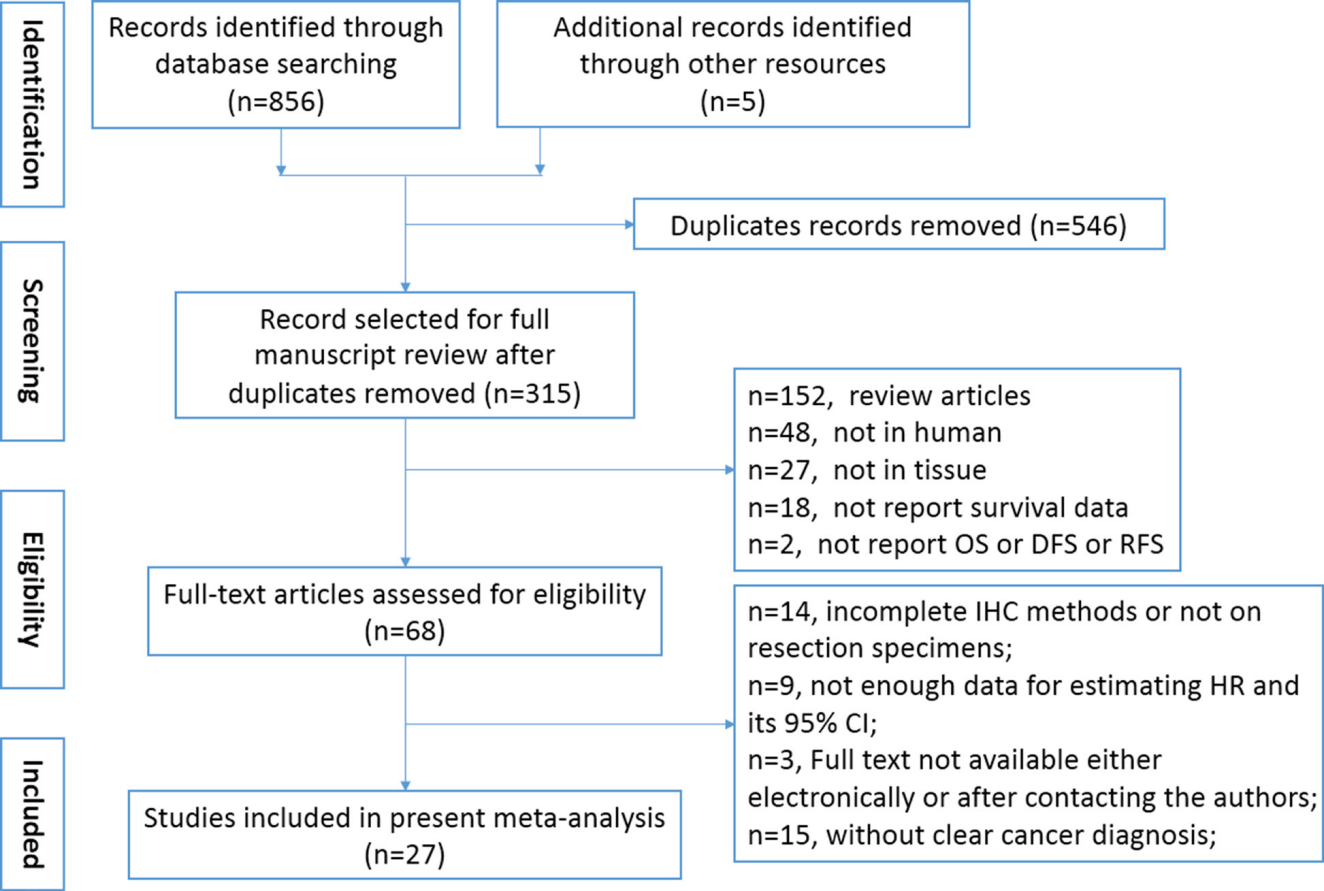
Flow chart of the selection of the studies in the meta-analysis

A total of 315 relevant studies were identified after removing duplicated records. The title and abstract of relevant articles were scrutinized by two authors (Yu and Chen) independently, and 247 citations were excluded from the first screening, leaving 68 citations for full-text review. After careful evaluation, only 27 studies with 4079 patients met the inclusion criteria for further analysis (Figure 1). The characteristics of the 27 included studies were shown in Table 1. Briefly, all eligible studies were retrospective studies that contained 49–617 samples and published from 2001 to 2016. All of the included studies measured the expression of GLUT1 by means of immunohistochemistry (IHC) staining or PCR in human tissues, and the cut-off values varied across studies. Among the studies, 4 evaluated lung cancer [5–8], 3 evaluated pancreatic cancer [9–11], 3 evaluated breast cancer [12, 14], 2 evaluated gallbladder cancer [10, 15], 2 evaluated gastric cancer [16, 17], 2 evaluated colorectal cancer [18, 19], 2 evaluated laryngeal cancer [20, 21], and 1 each evaluated hypopharyngeal cancer [22], endometrial cancer [23], salivary gland tumor [24], adrenocortical cancer [25], liver cancer [26], ampulla of Vater cancer [10], extrahepatic bile duct cancer [10], oral cancer [27], neuroblastic tumors [28], cervical cancer [29], ovarian cancer [30] and esophageal cancer [31]. Due to the retrospective design of the included studies, only five studies examined both OS and DFS [6, 12, 14, 25, 29]. Sixteen studies investigated the association between GLUT1 level and OS [7–11, 15–21, 24, 27, 28, 30], while four studies investigated the association between GLUT1 and DFS [5, 13, 23, 26]. Studies by Mineta [22] reported relapse-free survival data, whereas study by Sawayama [31] reported data of relapse-free survival and cancer-specific survival. Among the included studies, various antibodies were applied to evaluate the expression of GLUT1. Most of them were produced by Abcam and Dako, with dilution ranging 1:100 to 1:7500. The cut-off values varied dependent on staining score and the detection method. According to Newcastle-Ottawa Scale (NOS) tool, we systematically evaluated the quality of the included studies, and the results were shown in Supplementary Table 1. The included studies had a mean score of 7.2 (range 5 to 8), indicating the acceptable quality of included original studies.

**Table 1:** Characteristics of studies included in the present meta-analysis

Study	Country	Cancer types	Patient number	Recruitment time	Age	Follow-up months (median)	Method	Antibody source	Dilution	Cut-off	Positive rate(%)	Study Quality
Kawamura, 2011 [16]	Japan	Gastric cancer	617	1987–1989	27–88	NA	IHC	Polyclonal, Chemicon	1:4000	1.00%	29.5	6
Furudoi, 2001 [18]	Japan	Colorectal cancer	111	1983–1994	52.5–74.1	49.3–77.1 (mean = 63.2)	IHC	Polyclonal, DAKO	1:100	30%	35.1	7
Kang, 2002 [14]	Korea	Breast cancer	100	1996–1997	23–74	49–67 (median = 57.4)	IHC	Polyclonal, DAKO	1:200	10%	47	6
Mineta, 2002 [22]	Japan	Hypopharyngeal cancer	99	NA	39–94	6–192 (mean = 49)	IHC	Polyclonal, Chemicon	1:1000	70%	46.5	7
Sebastiani,2004 [23]	Italy	Endometrial cancer	87	1992–1996	27–92	Median = 60	IHC	Polyclonal, DAKO	NA	score=6	43	7
Mori, 2006 [24]	Japan	Salivary gland tumors	49	1990–2005	14–82	NA	IHC	Polyclonal, DAKO	1:50	15%	26.5	5
Lyshchik, 2007 [11]	Japan	Pancreatic cancer	74	NA	40–81	NA	IHC	Polyclonal, DAKO	1:200	60%	44.6	7
Legan, 2009 [15]	Slovenia	Gallbladder cancer	50	1998–2005	34–84	NA	IHC	Polyclonal, DAKO	1:100	50%	58	5
Fenske, 2009 [25]	Germany	Adrenocortical cancer	118	NA	NA	NA	IHC	Polyclonal, DAKO	1:100	10%	33.8	5
Kitamura, 2010 [26]	Japan	Liver cancer	63	2003–2005	32–80	2.5–66.7 (mean = 38)	IHC	Polyclonal, DAKO	1:200	0%	36.5	8
Sung, 2010 [10]	Korea	Gallbladder cancer	115	1983–2007	NA	1–160 (mean = 36)	IHC	Polyclonal, DAKO	1:200	5%	46.1	6
Sung, 2010 [10]	Korea	Pancreatic cancer	52	1983- 2007	NA	2–244 (mean = 28)	IHC	Polyclonal, DAKO	1:200	5%	51.9	6
Sung, 2010 [10]	Korea	Ampulla of Vater cancer	67	1983–2007	NA	1–264 (mean = 73)	IHC	Polyclonal, DAKO	1:200	= 5%	56.7	6
Sung, 2010 [10]	Korea	Extrahepatic bile duct cancer	121	1983- 2007	NA	1–235 (mean = 45)	IHC	Polyclonal, DAKO	1:200	= 5%	31.4	6
Andersen, 2011 [5]	Norway	Lung cancer	108	1990–2004	28–85	48–216 (median = 86)	IHC	Monoclonal, Abcam	1:500	25%	58.4	8
Jang, 2012 [12]	Korea	Breast cancer	276	2000–2009.	Mean= 50	NA (mean = 60)	IHC	Monoclonal, Abcam	1:250	10%	37.1	6
Sasaki, 2012 [7]	Japan	Lung cancer	279	2001–2008	29–86	NA	IHC	Monoclonal, Thermo Scientific	NA	NA	49.1	6
Kwon, 2013 [13]	Korea	Breast cancer	207	2000–2010	28–52.4	NA	IHC	Monoclonal, Abcam	1:200	10%	2.4	5
Maki, 2013 [6]	Japan	Lung cancer	105	2004–2006	29–83	NA (median = 59.7)	IHC	Monoclonal, Abcam	1:200	10%	26.7	8
Grimm, 2013 [27]	USA	Oral cancer	161	NA	NA	NA (mean = 52.26)	IHC	Polyclonal, DAKO	1:100	10%	41.6	8
Ramani, 2013 [28]	UK	Neuroblastic tumors	96	1994–2011	0.001–16	15–195 (median = 86)	IHC	Polyclonal,Merck-Millipore	NA	NA	45.8	8
Kim, 2013 [29]	Korea	Cervical cancer	162	1996–2010	NA	6–60 (mean = 55.6)	IHC	Monoclonal, NeoMarkers	1:3000	score = 8	22.8	7
Cho, 2013 [30]	Korea	Ovarian cancer	50	2008–2010	NA	NA (mean = 31.6)	IHC	Monoclonal, R&D Systems	NA	score = 3.85	52	7
Sawayama, 2014 [31]	Japan	Esophageal cancer	145	2000–2008	NA	1.3–132.3 (median = 39.5)	IHC	Polyclonal, Abcam	1:7500	50%	28.3	8
Yu, 2015 [9]	China	Pancreatic cancer	106	2000–012	31–77	NA	IHC	Monoclonal, Epitomics	1:250	score = 2	58.5	8
Osugi, 2015 [8]	Japan	Lung cancer	134	1998–2000	48–87	NA	IHC	Polyclonal, DAKO	1:500	50%	56	5
Starska, 2015 [20]	Poland	Laryngeal cancer	106	2003–2011	62.4 ± 9.1	NA	PCR	NA	NA	NA	83.9	6
Hans, 2015 [17]	Germany	Gastric cancer	150	2006–2011	NA	NA (mean = 33.2)	IHC	NA	1:100	= 10%	22	7
Goos, 2015 [19]	Netherlands	Colorectal cancer	214	1990–2010	NA	NA	IHC	Polyclonal, Abcam	1:600	NA	50	8
Zuo, 2016 [21]	China	Laryngeal cancer	57	2012–2014	NA	NA	IHC	NA, Epitomics	NA	NA	NA	5

Abbreviations: NA, not available; IHC, immunohistochemistery; WB, western blotting; TMA, tissue microarrayers.

### Prognostic value of GLUT1

A total of 232 patients in three studies [9–11] were included for evaluating the prognostic value of GLUT1 in pancreatic cancer. Results suggested that high expression of GLUT1 was associated with shorter overall survival in pancreatic cancer (fixed-effect model; HR = 1.469, 95% CI = 1.134–1.903, *p* = 0.004; *I*^2^ = 0%, *p* = 0.624). As for lung cancer [6–8] in three studies involved 518 patients, the pooled results suggested overexpression of GLUT1 had a significantly poor survival effect on OS (fixed-effect model; HR=2.188, 95% CI=1.348–3.553, *p* = 0.002; *I*^2^ = 0%, *p* = 0.685).

Since only a small part of studies reported other cancers, we just presented the qualitative summary and gave up quantitative synthesis. Some studies indicated that there was a poor prognostic value of GLUT1 in oral squamous cell carcinoma [27], malignant salivary gland tumors [24], colorectal cancer [18, 19], gastric cancer [16, 17], gallbladder cancer [10, 15], extrahepatic bile duct cancer [10], adrenocortical carcinoma [25] and neuroblastic tumor [28]. Others studies found there was no significant association between GLUT1 expression with prognosis in laryngeal cancer [20, 21], ampulla of vater cancer [10], extrahepatic bile duct cancer [10], cervical cancer [29], and ovarian cancer [2]. As for breast cancer, Kang [14] found there was no significant association between GLUT1 expression with OS, while Jang [12] identified a poor prognostic value of GLUT1 in their studies.

**Figure 2:**
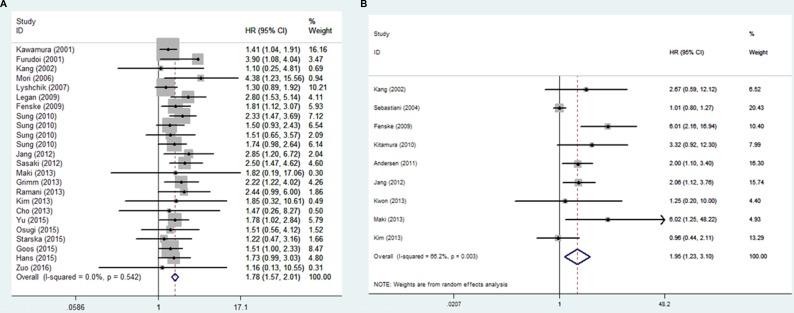
Forest plot of hazard ratio (HR) for the association between GLUT1 expression and OS (**A**) and DFS(**B**)

Among the studies reporting the OS data, the pooled results indicated that overexpression of GLUT1 was significantly associated with unfavorable OS (HR = 1.780, 95% CI = 1.574–2.013, *p <* 0.001). No significant heterogeneity was observed and the fixed-effect model was applied (I^2^ = 0.00%, *p* = 0.542) (Figure 2A).

There were nine studies reporting the DFS data [5, 6, 12–14, 23, 25, 26, 29]. Results showed that overexpression of GLUT1 was significantly associated with unfavorable DFS (HR=1.950, 95% CI =1.229–3.095, *p* = 0.003). There was statistically significant heterogeneity across the studies and the random-effect model was applied (I^2^ = 66.2%, *p* = 0.005) (Figure 2B).

Only two studies reported the RFS data, and qualitative summary was present. Study from Sawayama [31] found that overexpression of GLUT1 was associated with poor RFS in esophageal squamous cell carcinoma, whereas Mineta [22] found no significant association between GLUT1 and RFS in hypopharyngeal cancer. Besides, study from Sawayama [31] also indicated that overexpression of GLUT1 showed a significant disadvantage for esophageal cancer-specific survival.

### Correlation of GLUT1 expression with clinicopathological features

**Figure 3:**
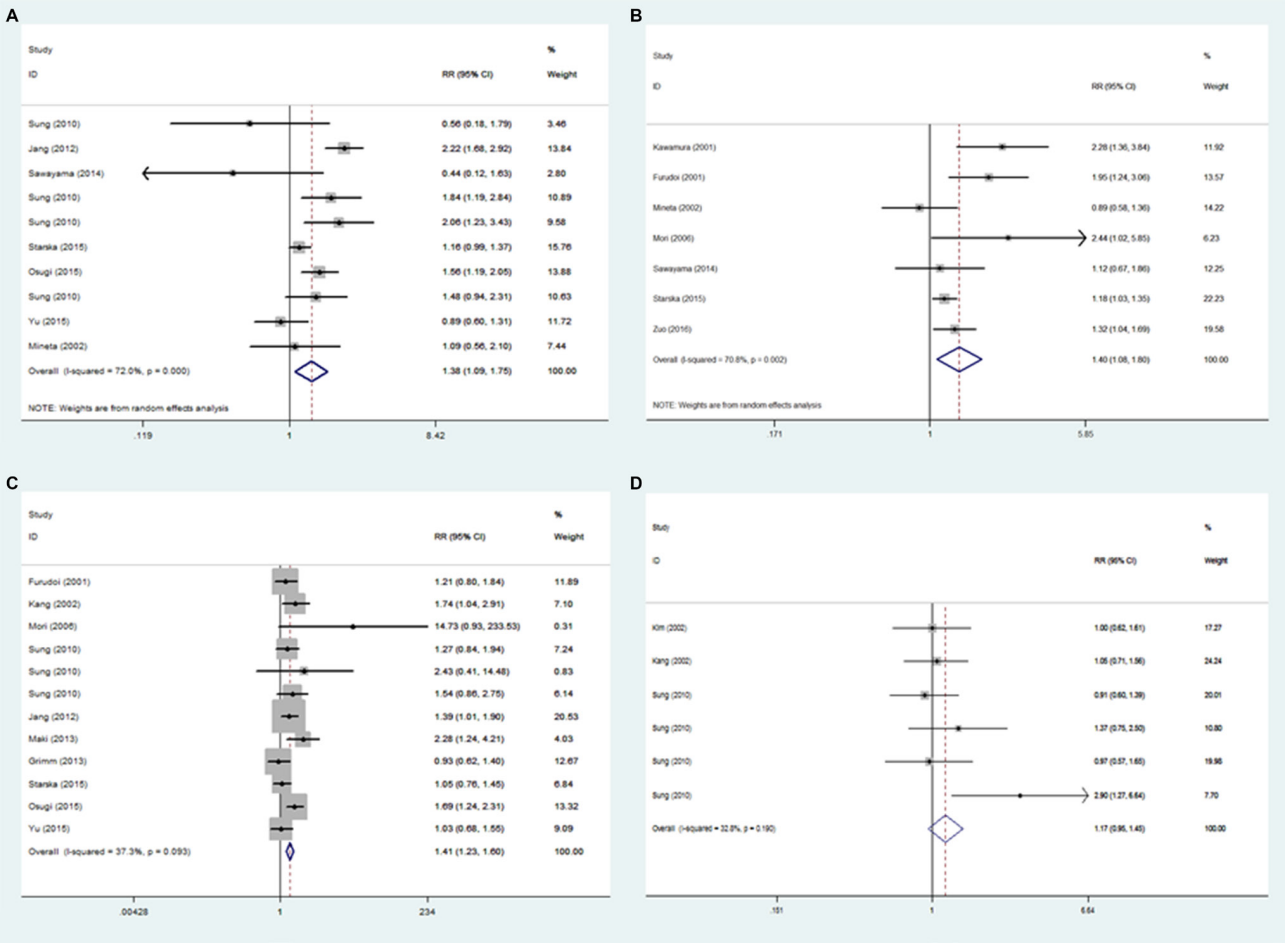
Forest plot of hazard ratio (HR) for the association between GLUT1 expression and characteristics parameters: poor differentiated tumors (**A**), positive lymph node metastasis (**B**), larger tumor size (**C**) and abnormal expression of p53 (**D**).

As shown in Figure 3, overexpression of GLUT1 was identified to be significantly associated with poor differentiated tumors (RR = 1.380, 95% CI = 1.086 – 1.755, *p* = 0.009; I^2^ = 72.0%, *p* < 0.001) (Figure 3A), positive lymph node metastasis (RR = 1.395, 95% CI = 1.082–1.799, *p* = 0.010; I^2^ = 70.8%, *p* = 0.002) (Figure 3B) and larger tumor size (RR = 1.405, 95% CI = 1.231 – 1.603, *p <* 0.001; I^2^ = 37.3%, *p* = 0.093) (Figure 3C). The overexpression of GLUT1 did not appear to be associated with age (RR = 1.063, 95% CI = 0.959–1.178, *p* = 0.244; I^2^ = 0.0%, *p* = 0.637) (Supplementary Figure 1A) and gender (RR = 1.196, 95% CI = 0.977–1.464, *p* = 0.083; I^2^ = 60.5%, *p* = 0.002) (Supplementary Figure 1B). In addition, overexpression of GLUT1 was more likely to be found in tumors with abnormal expression of p53. However, no significant relationship was identified in the pooled results (RR = 1.174, 95% CI = 0.953–1.448, *p* = 0.132; I^2^ = 32.8%, *p* = 0.190) (Figure 3D ).

### Heterogeneity

To explore the potential source of heterogeneity found in these analysis, subgroup analysis and sensitivity analysis were performed. Exploratory subgroup analyses were conducted according to ethnicity, sample size, cancer types, recruitment time, antibody source, methods of positive GLUT1 evaluation, study quality and cut-off value in OS. As indicated in Table 2, these variables did not alter the prognostic value of GLUT1 in OS. Interestingly, the prognostic impact of GLUT1 was numerically higher in the group of studies with larger sample size (> 100) (HR= 1.828, 95% CI = 1.583–2.111, *p <* 0.001), group of studies on non-gastrointestinal cancer (HR=2.132, 95% CI = 1.607–2.828, *p <* 0.001), group of studies using Dako antibody (HR = 1.927, 95% CI = 1.573–2.360, *p <* 0.001), group of studies using high cut-off value (range = 10%–100%) (HR = 2.325, 95% CI = 1.365-3.960, *p* = 0.002).

**Table 2:** Subgroup analyses for overall survival (OS) and disease-free survival (DFS)

Outcome		Characteristics	Number of studies	I-square	Hazard Ratio (95% confidence interval)	Lower CI	Upper CI	*P* value
OS	**Ethnicity**	**Between groups**						
		Caucasian	7	0.00%	1.859	1.492	2.318	< 0.001
		Asian	14	7.20%	1.771	1.51	2.078	< 0.001
	**Sample size**	Between groups*						
		< 100	8	0.00%	1.658	1.31	2.098	< 0.001
		> 100	14	0.00%	1.828	1.583	2.111	< 0.001
	**Cancer types**	**Between groups**						
		Gastrointestinal cancer	11	17.80%	1.738	1.488	2.031	< 0.001
		other cancers	10	0.00%	2.132	1.607	2.828	< 0.001
	**Recruitment time (Starting time)**	**Between groups**						
		Before 2000	10	21.20%	1.865	1.538	2.262	< 0.001
		After 2000	8	0.00%	1.922	1.461	2.53	< 0.001
		Others	3	20.20%	1.628	1.194	2.22	0.002
	**Antibody source**	**Between groups**						
		Dako	9	24.80%	1.927	1.573	2.36	< 0.001
		Abcam	3	0.00%	1.715	1.18	2.492	0.005
		others	9	0.00%	1.657	1.351	2.033	< 0.001
	**Evaluation of positive GLUT1 expression**	**Between groups**						
		Percentage of positive cells	12	21.80%	1.819	1.528	2.166	< 0.001
		Combination of intensity and percentage score	3	0.00%	1.716	1.099	2.821	0.019
		Others	6	0.00%	1.863	1.426	2.433	< 0.001
	**Study quality**	**Between groups**						
		≥ 7	10	6.20%	1.775	1.574	2.013	< 0.001
		< 7	11	0.00%	1.796	1.533	2.105	< 0.001
	**Cut-off value**	**Between groups**						
		Low level (range = 0%–10%)	8	0.00%	1.72	1.46	2.027	< 0.001
		High level (range = 10%–100%)	5	65.80%	2.325	1.365	3.96	0.002
		Others	8	0.00%	1.78	1.574	2.013	< 0.001
DFS	**Ethnicity**	**Between groups**						
		Asian	6	10.30%	1.871	1.186	2.951	0.007
		Caucasian	3	86.40%	2.026	0.856	4.794	0.108
	**Sample size**	**Between groups**						
		< 100	3	56.30%	1.657	0.702	3.913	0.249
		> 100	6	46.70%	2.141	1.286	3.565	0.003
	**Cancer types**	**Between groups**						
		Gastrointestinal cancer	1	NA	3.32	0.908	12.139	0.07
		other cancers	8	67.70%	1.86	1.151	3.005	0.011
	**Recruitment time**	**Between groups**						
		Start before 2000	4	51.90%	1.298	0.837	2.013	0.243
		Start after 2000	4	0.00%	2.326	1.401	3.861	0.001
		Others	1	NA	6.01	2.146	16.831	0.001
	**Detection methods**	**Between groups**						
		IHC only						
		IHC +TMA, IHC+WB						
	**Antibody source**	**Between groups**						
		Dako	4	79.80%	2.494	0.882	7.051	0.085
		Abcam	4	0.00%	2.091	1.41	3.101	0
		others	1	NA	0.96	0.438	2.102	0.919
	**Evaluation of positive GLUT1 expression**	**Between groups**						
		Percentage of positive cells	7	0.00%	2.463	1.745	3.477	0
		Combination of intensity and percentage score	2	0.00%	1.006	0.806	1.256	0.958
	**Study quality**	**Between groups**						
		≥ 7	5	63.10%	1.544	0.916	2.602	0.103
		< 7	4	17.90%	2.685	1.5	4.805	0.001
	**Cut-off value**	**Between groups**						
		Low level (range = 0%–10%)	6	0.00%	2.788	1.804	4.309	0
		High level (range = 10%–100%)	1	NA	2	1.138	3.516	0.016
		Others	2	0.00%	1.006	0.806	1.256	0.958

Subgroup analyses were also carried out to explore source of heterogeneity in DFS. As shown in Table 2, the prognostic value of GLUT1 in DFS was worse with respect to Asian group (HR = 1.871, 95% CI = 1.186–2.951, *p* = 0.007), larger sample size (HR = 2.141, 95% CI = 1.286–3.565, *p* = 0.007), late recruitment time (HR = 2.326, 95% CI = 1.401–3.861, *p* = 0.001), antibody produced by Abcam (HR = 2.091, 95% CI = 1.410–3.101, *p* < 0.001) and low study quality (HR = 2.685, 95% CI = 1.500–4.805, *p* = 0.001). The prognostic value of GLUT1 in DFS was also altered based on cancer types, methods of positive GLUT1 evaluation and cut-off values. However, these results needed to be interpreted cautiously because of the small number of eligible studies.

To gauge the stability of the results, sensitivity analysis was performed by assessing the potential impact of individual study on pooled data. As shown Table 3, the pooled results of OS and DFS was not significantly altered after exclusion of any study, indicating the robustness of present results (Supplementary Figure 2A and 2B).

**Table 3:** The influence of individual study on the pooled estimate for outcomes

Outcome	Study omitted	Estimate	[95% confidence interval]	
OS	Kawamura, 2011 [19]	1.862	1.628	2.129
	Furudoi, 2001 [21]	1.731	1.527	1.961
	Kang, 2002 [17]	1.786	1.579	2.020
	Mori, 2006 [27]	1.765	1.560	1.997
	Lyshchik, 2007 [14]	1.845	1.621	2.100
	Legan, 2009 [18]	1.746	1.540	1.979
	Fenske, 2009 [28]	1.778	1.567	2.018
	Sung, 2010 [13]	1.744	1.535	1.981
	Sung, 2010 [13]	1.802	1.587	2.046
	Sung, 2010 [13]	1.786	1.578	2.022
	Sung, 2010 [13]	1.783	1.571	2.024
	Jang, 2012 [15]	1.763	1.557	1.996
	Sasaki, 2012 [10]	1.751	1.544	1.986
	Maki, 2013 [9]	1.780	1.574	2.013
	Grimm, 2013 [30]	1.763	1.555	1.999
	Ramani, 2013 [31]	1.770	1.563	2.003
	Kim, 2013 [32]	1.780	1.574	2.013
	Cho, 2013 [33]	1.782	1.575	2.015
	Yu, 2015 [12]	1.780	1.569	2.020
	Osugi, 2015 [11]	1.785	1.577	2.020
	Starska, 2015 [23]	1.792	1.583	2.028
	Goos, 2015 [22]	1.807	1.589	2.054
	Hans, 2015 [20]	1.783	1.572	2.022
	Zuo, 2016 [24]	1.783	1.576	2.016
	Combined	1.780	1.574	2.013
DFS	Kang, 2002 [17]	1.916	1.179	3.114
	Sebastiani, 2004 [26]	2.218	1.466	3.354
	Fenske, 2009 [28]	1.633	1.087	2.453
	Kitamura, 2010 [29]	1.860	1.151	3.005
	Andersen, 2011 [8]	1.986	1.156	3.413
	Jang, 2012 [15]	1.968	1.155	3.353
	Kwon, 2013 [16]	2.008	1.235	3.264
	Maki, 2013 [9]	1.828	1.153	2.897
	Kim, 2013 [32]	2.214	1.308	3.747
	Combined	1.950	1.229	3.095

### Publication bias

**Figure 4:**
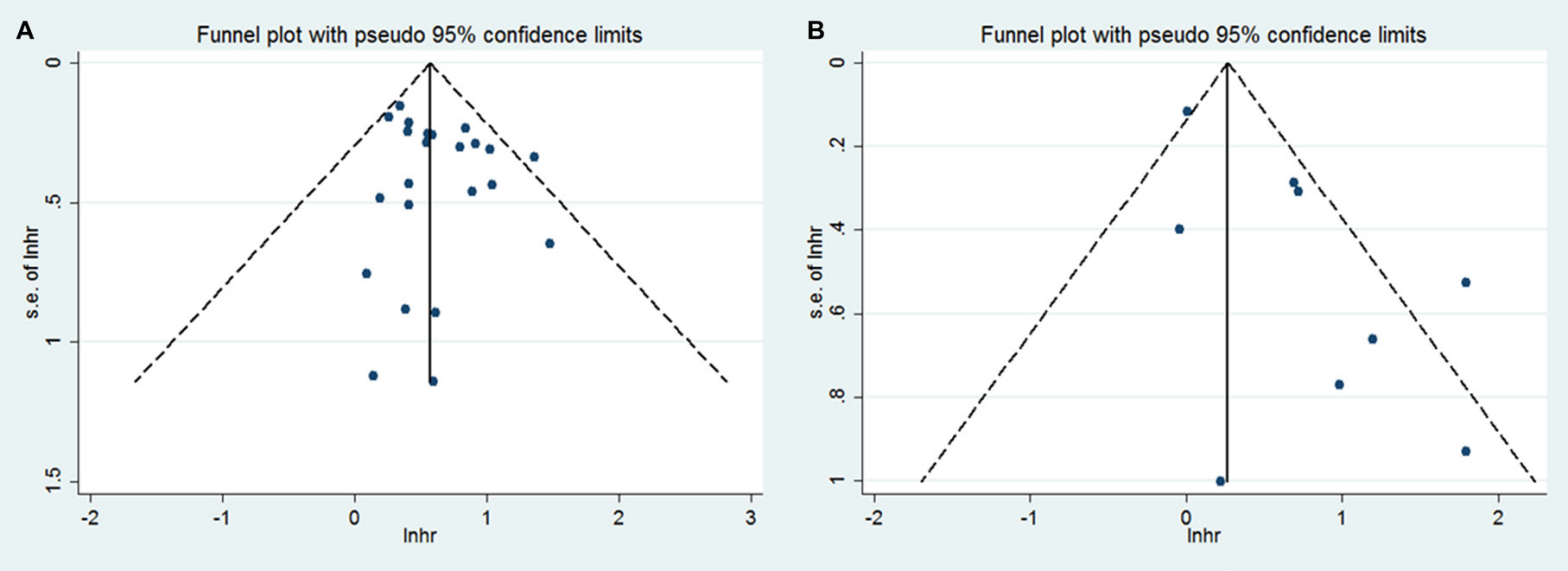
Funnel plot for the assessment of publication bias in this study (**A**) Funnel plot for 21 studies reporting overall survival. (**B**) Funnel plot for 9 studies reporting disease-free survival.

We assessed the publication bias by visually assessing a funnel plot for asymmetry and by quantitatively performing Begg's test and Egger's test. The funnel plots showed evidence for symmetry in both OS (Figure 4A) and DFS (Figure 4B). No publication bias was found in the overall survival meta-analysis (Begg's test, *p* = 0.359; Egger's test, *p* = 0.207). The Egger's test was significant (*p* = 0.022) for publication bias but not the Begg's test (*p* = 0.917) in the disease-free survival meta-analysis. Considering the non-normal distribution of the included patient numbers and the discrepancies of these two tests, the Egger test is not to be trusted. Therefore, there is no significant publication bias in the above analyses (Figure 4). The finding was another strong evidence to verify that GLUT1 was an independent prognostic factor in various tumors.

## DISCUSSION

Overexpression of GLUT1 may represent a key mechanism by which malignant cells may achieve increased glucose uptake and compensate the lack of energy caused by inefficient anaerobic glycolysis [2]. Therefore, the prognostic value of GLUT1 have been extensively explored in various cancers [5–31]. However, inconsistent results were found in different studies [12, 14, 29]. So far, there is no meta-analysis regarding the association between GLUT1 expression and survival of tumors. To provide comprehensive and reliable conclusions, we conducted the present meta-analysis to assess the prognostic value of GLUT1 in tumors. Our comprehensive meta-analysis of 4079 patients included in 27 different studies indicated that overexpression of GLUT1 associated with worse OS and DFS. Moreover, we provided evidence that abnormal expression of GLUT1 was significantly associated with poor differentiated tumors, positive lymph node metastasis and larger tumor size, which suggested that overexpression of GLUT1 linked with enhanced invasive potential, proliferative activity, and decreased patient survival. Subgroup analyses were performed to explore the source of heterogeneity based on ethnicity, sample size, cancer types, recruitment time, antibody source, methods of positive GLUT1 evaluation, study quality and cut-off value. We found that these variables did not alter the prognostic value of GLUT1 in OS, whereas prognostic value of GLUT1 in DFS was more obvious in Asian group, larger sample size, late recruitment time, antibody produced by Abcam and low study quality. In light of these findings, we hypothesized that GLUT1 may contribute to the pathogenesis of cancers. Therefore, GLUT1 may be an ideal prognostic factor in various cancers.

However, the mechanism how GLUT1 contributes to the oncogenesis remains unclear. Previous studies have pointed out that significantly higher GLUT1 mRNA expression levels were identified in various cancer tissues and cell lines compared to normal cells and matched non-tumor tissue. Further suppression of GLUT1 expression significantly impaired both the survival and migratory potential of cancer cells. Moreover, inhibition of GLUT1 chemosensitized head and neck cancer cells to cisplatin [32–35]. Recent studies found that GLUT1 overexpression significantly upregulated the expression of NFκB-p65, and it was reversed by inhibition of GLUT1 expression [33]. Given the oncogenic role of NFκB-p65 in tumorigenicity, the survival effects of GLUT1 may be associated with the activation of the NFκB pathway [36]. Recent study identified that translocation of GLUT1 onto the plasma membrane from para-glogian area was dependent on activation of the PI3KC1-AKT pathway. The results suggested that overexpression of GLUT1 in proliferating cancer cells was associated with the abnormal activation of the PI3KC1-AKT pathway, consequent to the mutational activation of PI3KC1 and/or the loss of PTEN [37]. In addition, several signaling molecules and pathways were showed to be involved in the regulation of expression and distribution of GLUT1, such as hypoxia induced factor 1, c-Myc, Ras and p53 signaling pathway [38], which suggested that signaling network was really complex in regulation of GLUT1. Further elucidation of signaling network of GLUT1 may provide novel methods for detection and treatment of cancer. Currently, GLUT1 expression could be measured simply and inexpensively as part of the routine histologic biopsy of tumors samples prior to operation [9]. The present results may vary from other meta analyses with respect to colorectal cancer, breast cancer and oral squamous cell carcinoma, which is partly attributed to different inclusion criteria and different research Interests [39–43]. Unlike other meta-analyses, our present analysis not only assess the association between GLUT1 and nineteen kinds of cancers, but also employed HR to assess the impact of GLUT1 on survival. The number of eligible studies is small because of the strict inclusion criteria. However, the quality of the included study and the reliability of present results were guaranteed. Evidence showed that it is not suitable to use OR or RR in a meta-analysis of time-to-event outcomes. Those dichotomous measures can result in combining trials reported at different stages of maturity, with variable follow up, resulting in an estimate that is both unreliable and difficult to interpret [44]. Therefore, we applied HR to estimate the prognostic value of GLUT1 in various cancers. According to the results in present analysis, GLUT1 has an ideal prognostic value in various cancers, and the feasible histologic biopsy may be helpful for more adequate clinical decision.

Certainly, further studies are essential to confirm the credibility of our result. Some important limitations of this meta-analysis should be considered when interpreting the results. Firstly, only papers published in English was included, which probably introduced bias. Secondly, different methods of survival data analysis in different studies should be considered as a potential source of heterogeneity. Although most studies adjusted their HRs and 95% CIs using multivariate analysis, variables added into Cox proportional hazard models were different from study to study. Thirdly, GLUT1 staining was heterogeneous, cytoplasmic and membranous. Most of the included studies did not evaluate the cytoplasmic and membranous staining separately. Therefore, the individual prognostic value of cytoplasmic and membranous GLUT1 remains further evaluation. Finally, metabolism is a complex, multistep, and highly dynamic process. Therefore, a prognostic model integrating a group of valuable metabolic markers may be more accurate in predicting cancer prognosis.

## CONCLUSIONS

To sum up, this systematic review and meta-analysis indicated that a low expression of the GLUT1 predicted favorable prognosis in gallbladder cancer, pancreatic cancer, breast cancer, and lung cancer. However, due to the limitations in terms of quality and quantity of published original articles, the clinical utility of this biomarker is still reliant on future validation. Therefore, more high-quality, large-sample, prospectively designed studies are highly recommended.

## MATERIALS AND METHODS

### Publication search strategy and selection criteria

Up to May 2016, a systematic search was conducted using PubMed, Medline, Springer and Cochrane library. We identified articles using the following search strategies: (cancer OR carcinoma OR neoplasm OR tumor) AND (prognos* OR surviv*) AND (GLUT1 OR Glucose transporter-1 OR SLC2A1). Studies included in our study had to meet the following criteria: (1) hazard ratio (HR) and 95% confidence interval (CI) for overall survival (OS)/disease-free survival (DFS)/relapse-free survival(RFS)were reported or could be extracted from data presented; (2) when the same group of patients was reported in multiple studies, the most informative one was included; (3) availability of full papers in English; (4) assessment of the expression of GLUT1 in human tissues and the sample size of the study was more than 40 patients. The exclusion criteria were as follows: (1) literature reviews, comments, letters, or duplicated publications; (2) no sufficient data to estimate the HR and 95% CI; or (3) the full text could not be retrieved even if the contact with authors had been made.

### Data extraction and quality assessment

Two authors (Yu and Chen) carefully read the full texts independently and extracted the data to avoid bias in the process of data-abstraction. The following information was recorded: the first author's name, the country of authors, the year of publication, cancer types, patient number, age, follow-up months, detection method, primary antibody, dilution concentration, cut-off value, positive rate and so on. The Newcastle-Ottawa Scale was applied to assess the quality of each included study. The NOS criteria was scored based on three aspects: (1) subject selection, (2) comparability of subject, (3) clinical outcome. Scores based on NOS of 1–3, 4–6, and 7–9 were defined as low-, intermediate-, and high-quality studies, respectively. All disagreements were discussed and resolved with consensus.

### Statistical analysis

Meta-analysis was performed using the Stata (version 11 for Windows). For the quantitative aggregation of the survival results, HRs and their 95% CIs were used. In some studies, where HRs and corresponding 95% CIs of low expression versus high expression were provided, we calculated reciprocal to get high expression versus low expression data. In studies where HRs and corresponding 95% CIs were not directly reported, we estimated these values on the basis of available data, such as survival curves, using the methods developed by Parmar [45], Williamson [46], and Tierney [44]. When analyzing the relationship between GLUT1 and clinicopathological factors, risk ratios (RR) and their 95% CI were applied. Heterogeneity was assessed by the Chi-squared test and *p value* in our meta-analysis. I^2^ value was used to evaluate the heterogeneity, fixed-effect model was used if there was I^2^ = 0–50%, which means no significant heterogeneity. Otherwise, the random-effects model was applied. Forest plots were used to illustrate the HRs and 95% CIs of each included study and the results were pooled. To visually assess the possibility of publication bias in a meta-analysis, we produced a funnel plot of the estimated effects. Further Egger's test and Begg's test were performed to weigh the potential publication bias. Sensitivity analysis was performed by extraction of each single study to investigate the stability of the results. All *p* values were two-side, being statistically significant when *p value* less than 0.05. As for only two studies were focused on RFS data, we just presented the qualitative summary and gave up quantitative synthesis.

## SUPPLEMENTARY FIGURES AND TABLE


