# Supplementary material for: The prognostic value of GLUT1 in cancers: a systematic review and meta-analysis
Source: Oncotarget. 2017 Apr 27;8(26):43356–67. doi: 10.18632/oncotarget.17445 (PMC5522151; doi:10.18632/oncotarget.17445)
Supplement: Supplementary file 1 [file oncotarget-08-43356-s001.pdf]

The prognostic value of GLUT1 in cancers: a systematic review and meta-analysis

Supplementary Materials

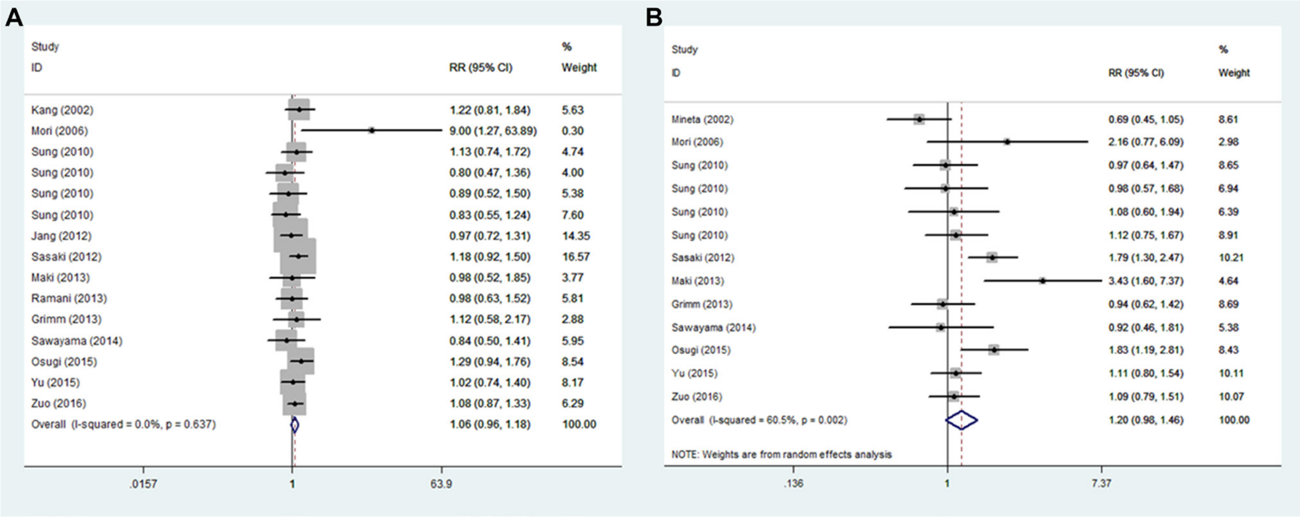

Supplementary Figure 1: Forest plot of hazard ratio (HR) for the association between GLUT1 expression and characteristics parameters: age (**A**) gender (**B**).

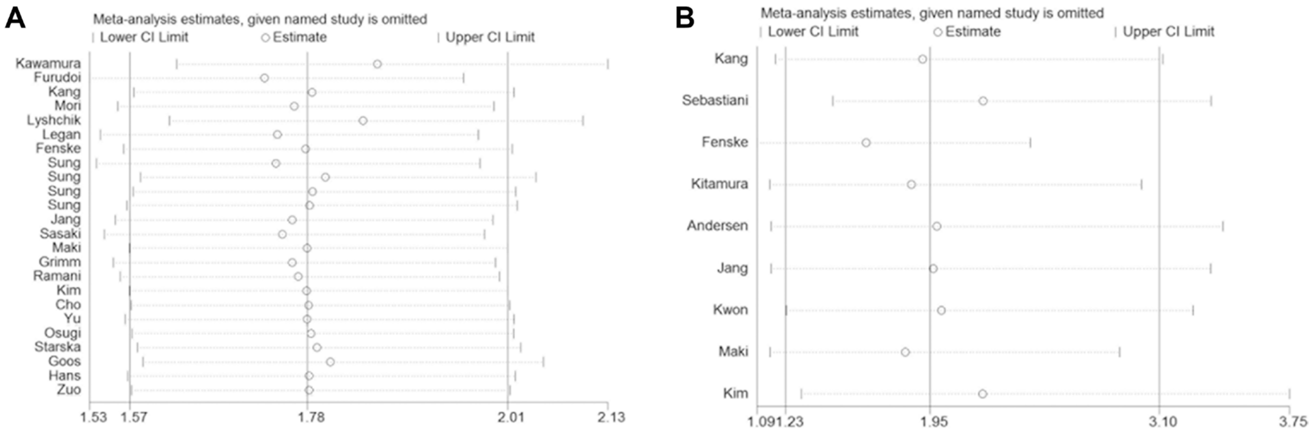

Supplementary Figure 2: Sensitivity analysis were performed in studies reporting association of GLUT1 expression and OS (**A**) DFS (**B**).

Supplementary Table 1: Methodological quality of all studies based on the newcastle-ottawa scale

Author	Representativeness of exposed cohort	Selection of non-exposed cohort	Assessment of exposure	Outcome not present at start of study	Comparability based on the design or analysis	Assessment of outcome	Follow-up long enough for outcomes	Adequacy of follow-up	Total score
Kawamura, 2001	1	1	1	1	1	1	0	0	6
Furudoi, 2001	1	1	1	1	0	1	1	1	7
Kang, 2002	1	1	1	1	0	1	1	0	6
Mineta, 2002	1	1	1	1	1	1	1	0	7
Sebastiani, 2004	1	1	1	1	0	1	1	1	7
Mori, 2006	1	1	1	1	0	1	0	0	5
Lyshchik, 2007	1	1	1	1	0	1	1	1	7
Legan, 2009	1	1	1	1	0	1	0	0	5
Fenske, 2009	1	1	1	1	0	1	0	0	5
Kitamura, 2010	1	1	1	1	1	1	1	1	8
Sung, 2010	1	1	1	1	0	1	1	0	6
Sung, 2010	1	1	1	1	0	1	1	0	6
Sung, 2010	1	1	1	1	0	1	1	0	6
Sung, 2010	1	1	1	1	0	1	1	0	6
Andersen, 2011	1	1	1	1	1	1	1	1	8
Jang, 2012	1	1	1	1	0	1	1	0	6
Sasaki, 2012	1	1	1	1	0	1	1	0	6
Kwon, 2013	1	1	1	1	0	1	0	0	5
Maki, 2013	1	1	1	1	1	1	1	1	8
Grimm, 2013	1	1	1	1	1	1	1	1	8
Ramani, 2013	1	1	1	1	1	1	1	1	8
Kim, 2013	1	1	1	1	0	1	1	1	7
Cho, 2013	1	1	1	1	1	1	1	0	7
Sawayama, 2014	1	1	1	1	1	1	1	1	8
Yu, 2015	1	1	1	1	1	1	1	1	8
Osugi, 2015	1	1	1	1	0	1	0	0	5
Starska, 2015	1	1	1	1	1	1	0	0	6
Hans, 2015	1	1	1	1	0	1	1	1	7
Goos, 2015	1	1	1	1	1	1	1	1	8
Zuo, 2016	1	1	1	1	0	1	0	0	5
